# Distinct Effects of PFOS and OBS on Neurotoxicity via PMK-1 Mediated Pathway in *Caenorhabditis elegans*

**DOI:** 10.3390/toxics13080662

**Published:** 2025-08-06

**Authors:** Jiahong Jiang, Qi Liu, Boxiang Zhang, Lei Zhao, Dan Xu

**Affiliations:** Institute of Environmental Systems Biology, College of Environment Science and Engineering, Dalian Maritime University, Linghai Road 1, Dalian 116026, China; 18390805822@163.com (J.J.); liuqi127140@163.com (Q.L.); bxzhang@dlmu.edu.cn (B.Z.); zhaol@dlmu.edu.cn (L.Z.)

**Keywords:** PFOS, OBS, *C. elegans*, neurotoxicity, PMK-1

## Abstract

Sodium p-perfluorous nonenoxybenzenesulfonate (OBS) has been proposed as a substitute for perfluorooctanesulfonic acid (PFOS), yet it has garnered increasing attention due to its environmental persistence and potential toxicity. Despite these concerns, the neurotoxic mechanisms of OBS remain unclear. This study investigates and compares the neurotoxic effects and mechanisms of OBS and PFOS in *Caenorhabditis elegans*. L4-stage worms were exposed to OBS (0.1–100 μM) or PFOS (100 μM) for 24 h. Neurobehavioral analysis showed that OBS exposure induced concentration-dependent neurobehavioral deficits, with 100 μM OBS significantly reducing pharyngeal pumping rate (29.8%), head swing frequency (23.4%), and body bending frequency (46.6%), surpassing the effects of PFOS. Both compounds decreased the fluorescence intensity of dopaminergic, glutamatergic, and γ-aminobutyric acid neurons and downregulated neurotransmitter-associated genes. They also increased ROS generation and inhibited antioxidant gene expression. Molecular docking revealed that OBS had a stronger binding affinity to p38 MAPK key protein (PMK-1) than PFOS. OBS and PFOS upregulated *pmk-1* and *skn-1*, modulating oxidative stress and neuronal function. *pmk-1* mutation differentially affected OBS-induced neurobehavioral changes and gene expression alterations. Our findings indicate that OBS exhibits stronger neurotoxicity than PFOS in *Caenorhabditis elegans*, mediated through the PMK-1 pathway. These results highlight the need for further investigation into the safety of OBS as a PFOS alternative.

## 1. Introduction

Pentafluorooctanesulfonate (PFOS), belongs to perfluoroalkyl and polyfluoroalkyl substances (PFASs), which is a globally pervasive environmental pollutant [1]. PFOS is widely utilized in firefighting foams, textile waterproofing treatments, leather processing, and electronic product manufacturing [2]. PFOS exhibits remarkable resistance to biodegradation and a propensity for bioaccumulation, with a half-life of approximately 5 years in the human body [3]. PFOS has been detected in various environmental media and enters human body through contaminated food, water, air, or skin contact [4,5]. It has been shown that PFOS adversely affects multiple organ systems, including the liver, immune system, and endocrine system [6]. Moreover, its neurotoxic effects are increasingly recognized, with studies demonstrating that PFOS can induce oxidative stress and affect dopaminergic neurons, leading to behavioral abnormalities, cognitive dysfunction, and neurodegenerative diseases [7].

Sodium p-perfluorous nonenoxybenzenesulfonate (OBS) is a typical substitute for PFOS, which are primarily used in firefighting foams and oil recovery [8]. Similar with PFOS, OBS exhibits significant hydrophobicity and chemical stability. Despite its reduced bioaccumulative properties, OBS demonstrates high persistence in the environment, accumulating in water bodies, sediments, and organisms [9]. In lakes and sediments surrounding the Daqing Oilfield, OBS detection rates are 91% and 100%, with average concentrations of 409.73 ng/L and 59.87 ng/g, respectively [10]. The average concentration of OBS in the blood of wild crucian carp is 144 μg/L, while in pregnant women’s serum, it is detected at 0.711 ng/mL [11]. OBS exhibits similar acute toxicity to PFOS, causing mortality in fish and tadpoles, with LC50 (96 h) values of 25.5 mg/L and 28.4 mg/L, respectively [10]. OBS has demonstrated developmental toxicity in fish, inducing oxidative stress in the livers of juvenile and adult zebrafish, disrupting circadian rhythms in adult zebrafish [11,12]. OBS is associated with metabolic disorders in mice, increased human obesity, decreased bone density, and cell necrosis and oxidative stress in HepG2 cells [13,14,15,16]. However, potential impacts of OBS on the nervous systems remains unknown.

*Caenorhabditis elegans (C. elegans)* are transparent C elegans with short lifespan and ease of cultivation, making model organism for studying the neurotoxicity of environmental pollutants [17]. Nervous system of *C. elegans* is structurally and functionally analogous to mammals, and neuronal damage is reflected in behavioral abnormalities [18,19]. It is reported that many environmental pollutants, including metals, pesticides, and organic compounds, exhibit neurotoxicity in *C. elegans*, with results often paralleling those in mammalian systems [20]. The transparency of *C. elegans* and its ease of genetic manipulation, such as with green fluorescent protein (GFP) fusion, enable visualization of cellular morphology and protein expression patterns in vivo [21]. GFP-labelled C. elegans often were utilized to study classical neurotransmitters such as dopamine (DA), glutamate (Glu), gamma-aminobutyric acid (GABA), and acetylcholine (ACh), which linked to neurodegenerative diseases like Alzheimer’s disease (AD) and Parkinson’s disease (PD), elucidating the pathogenesis and progression of these diseases [20].

It is known that p38 MAPK (mitogen-activated protein kinase) is a member of the MAPK family, which plays a crucial role in cellular stress responses [22]. In *C. elegans*, the homolog of p38 MAPK is PMK-1, which is the key kinase in p38 MAPK pathway [23]. When the nematode is exposed to oxidative stress, PMK-1 phosphorylates the transcription factor SKN-1 (the homolog of Nrf2), promoting its translocation to the nucleus, which is crucial for scavenging reactive oxygen species (ROS) and maintaining cellular homeostasis [24,25]. ROS regulates the expression of antioxidant genes such as *gpx-4*, *gst-4*, *sod-1*, and *sod-3* by activating SKN-1 [26,27]. Mutants of PMK-1, such as KU25 [pmk-1 (km25)] strain, are commonly studied variants where the function of the p38 MAPK pathway may be inhibited or altered in the nematode [28]. The pmk-1 mutant exhibits higher sensitivity to various stress conditions, such as heat stress, oxidative stress, and pathogen infection [29,30]. Therefore, the use of *pmk-1* mutant can help to further understand the role of PMK-1 in cellular stress responses.

In this study, we systematically investigated the potential neurotoxic effects and underlying mechanisms of exposure to OBS in comparison to PFOS in *C. elegans*. First, we analyzed the effects of OBS exposure on the neurobehavioral functions of C elegans and the dose–response relationship, and compared it with PFOS to clarify its toxic effects. Then, we explored the interference of PFOS/OBS exposure on oxidative stress and the neurotransmitter system in C elegans. We utilized three GFP-tagged transgenic strains of *C. elegans* to evaluate neuronal damage. Finally, we elucidated the key role of the p38 MAPK pathway in PFOS/OBS neurotoxicity, by comparing the behavioral, oxidative stress, and neurotransmitter changes in N2 wild-type and *pmk-1* mutant C elegans after PFOS/OBS exposure. This study provides an important basis for the scientific assessment of the neurotoxic risks of OBS as a substitute for PFOS.

## 2. Materials and Methods

### 2.1. C. elegans Strains and Culture 

The following strains of *C. elegans* were obtained from Caenorhabditis Genetics Center, including N2 (wild-type), BZ555 (dat-1p::GFP, dopaminergic neuron marker), DA1240 (eat-4::GFP), glutamatergic neuron marker), EG1285 (unc-47p::GFP, GABAergic neuron marker), KN259 (sod-3p::GFP, oxidative stress reporter), and KU25 [*pmk-1 (km25)*, p38 MAPK mutant]. Synchronized eggs were obtained by lysing gravid adults with sodium hypochlorite solution (5 M NaOH, NaClO) and then cultured on nematode growth medium (NGM) seeded with *Escherichia coli (E. coli)* OP50 as a food source. The worms were maintained in a sterile incubator at 20 °C in the dark for 48 h until they reached the L4 larval stage. The NGM culture medium was prepared with 3 g/L NaCl, 2.5 g/L peptone, 17 g/L agar, 1 mM CaCl_2_, 1 mM MgSO_4_, 25 mM potassium phosphate buffer (at a pH of 6.0) and 5 μg/mL cholesterol. M9 buffer consisted of 3 g/L K_2_HPO_4_, 6 g/L Na_2_HPO_4_, 5 g/LNaCl, and 0.5 mM MgSO_4._ The above various reagents were supplied by Sangon Biotech (Shanghai, China).

### 2.2. Exposure to PFOS and OBS

PFOS (CAS: 1763-23-1) and OBS (CAS: 70829-87-7) were purchased from Tokyo Chemical Industry Co., Ltd. (Tokyo, Japan) and Shanghai Futian Chemical Technology Co. (Shanghai, China), respectively. The average environmental concentration of OBS is 0.28 µM, with a concentration range of 2.11 × 10^−4^ M–6.13 µM [31]. Therefore, a concentration range of 0.1–10 µM OBS was selected to cover environmentally relevant concentrations to higher doses. Meanwhile, PFOS was used as a positive control because exposure to 100 µM PFOS can cause damage to the dopaminergic neurons of C elegans and alter the level of ROS accumulation [32], supporting its use as a positive control for neurotoxicity in our study.

*C. elegans* were exposed to different concentrations of OBS (0.1, 1, 10, and 100 μM) or PFOS (100 μM, positive control) for 24 h. 0.1% dimethyl sulfoxide (DMSO) was used as vehicle control. In 60 mm solid culture plates, 200 μL of exposure solution at the respective concentrations was added along with synchronized L4-stage *C. elegans*. The plates were then placed in a biochemical incubator (Lichen, Shaoxing, Zhejiang, China) for 1 day, with three biological replicates per group.

### 2.3. Neurobehavioral Activity Measurement

After exposure, the C elegans were washed with M9 buffer and transferred to NGM solid plates without food. They were allowed to recover for 1 min before being observed under a stereomicroscope (Mshot, Guangzhou, China). The number of head swings within 30 s was recorded, where one complete swing from left to right and back to left was counted as one event.

Subsequently, the exposed C elegans were transferred to NGM plates supplemented with *E. coli* OP50, and the number of pharyngeal pumping events within 20 s was recorded. Each upward and downward movement of the pharyngeal pump was counted as one event.

After wash with M9 buffer, the C elegans were placed on NGM solid plates without food for a 1 min recovery period. The number of body bends within 20 s was observed under a stereomicroscope. A body bend was defined as the movement of the nematode in a sinusoidal wave along its longitudinal axis. All experiments included three independent biological replicates, with 15 worms (*n* = 15) per group in each replicate.

### 2.4. Neuronal Damage Observation

The GFP-labelled strains BZ555, DA1240, and EG1285 were used to assess neuronal damage. Following exposure, these worms were slowly washed with M9 buffer and collected into 1.5 mL tubes. After allowing them to settle, the supernatant was discarded, and the washing procedure was repeated three times. Subsequently, 5 mM levamisole solution (Aladdin, Shanghai, China) was added to anesthetize the C elegans, and once they were immobilized, they were transferred to glass slides, and imaged under a fluorescence microscope (Nikon, Tokyo, Japan). The average fluorescence intensity of green fluorescent protein expression in the neurons of each strain was measured using ImageJ 2.2.0 software. All experiments included three independent biological replicates, with 20 worms (*n* = 20) per group in each replicate.

### 2.5. Reverse Transcription Quantitative PCR (RT-qPCR)

Total RNA was extracted using RNA Isolater reagent (Vazyme Biotech, Nanjing, China). RT-qPCR was performed using SupRealQ Ultra Hunter SYBR qPCR Master Mix (Vazyme Biotech) according to the manufacturer’s protocol. The tba-1 gene was used as a reference, and the relative expression levels were analyzed using the comparative 2^−ΔΔCT^ method. Primer sequences are provided in the Table S1. All experiments included three independent biological replicates, with 15 worms (*n* = 15) per group in each replicate.

### 2.6. Molecular Docking

The 2D structure of the small molecule ligand (OBS/PFOS) was retrieved from PubChem, converted to 3D using ChemOffice 20.0 with energy minimization (MMFF94), and saved as a.mol2 file, while the PMK1 target sequence (UniProtKB: Q17446) was modeled via AlphaFold and saved as a.pdb file. Using Molecular Operating Environment 2019 software, the protein was prepared (heteroatom removal, protonation at pH 7.4, AMBER10:EHT minimization) and active pockets were predicted, followed by molecular docking (50 runs, London dG scoring) with binding energy thresholds: ≥−4.25 (weak), <−5.0 (moderate), and <−7.0 kcal/mol (strong). Results were visualized in PyMOL 2.6.0 and Discovery Studio 2019, analyzing interaction patterns (H-bonds, hydrophobic contacts) and pose stability (RMSD clustering).

### 2.7. Correlation Analysis

The Mantel test is used to analyze the global correlation between gene expression profiles and phenotype feature matrices. It quantitatively evaluates the strength of gene-phenotype association by calculating the Pearson correlation coefficient (with a value range of −1 to 1) between the neuron gene expression matrix and the oxidative stress gene expression matrix. All calculations are implemented through the Vegan package (v2.6.10) in R language (R version 4.4.2).

### 2.8. Statistical Analysis

Data are presented as means ± SEM. Statistical analysis was performed using one-way ANOVA in GraphPad Prism9.5.0 (GraphPad Software, San Diego, CA, USA). Significant differences were determined by comparing the results of the experimental groups to those of the control group (* *p* < 0.05, ** *p* < 0.01 vs. DMSO control; ^#^ *p* < 0.05, ^##^ *p* < 0.01 vs. PFOS).

## 3. Results

### 3.1. Distinct Effects of PFOS/OBS Exposure on Neurobehavioral Activity in C. elegans

**Figure 1:**
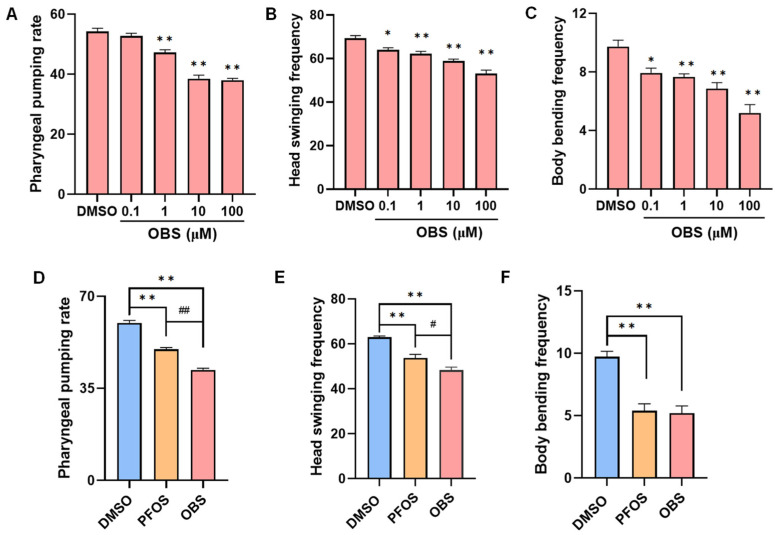
Effects of PFOS/OBS exposure on neurobehavioral activity in *C. elegans*. (**A**–**C**) The worms were treated with different concentrations of OBS or DMSO control, following by the measure of (**A**) Pharyngeal pumping rate, (**B**) Head swinging frequency, (**C**) Body bending frequency. (**D**–**F**) Alteration of neurobehavioral activity including (**D**) Pharyngeal pumping rate, (**E**) Head swinging frequency, and (**F**) Body bending frequency were compared when exposure to the same concentration (100 μM) of PFOS and OBS. * *p* < 0.05, ** *p* < 0.01 vs. DMSO. ^#^ *p* < 0.05, ^##^ *p* < 0.01 vs. PFOS. *n* = 15.

We investigated the effects of OBS exposure on neurobehavioral activity, including pharyngeal pumping rate, frequency of head swings and body bends in *C. elegans*. The results showed that the pharyngeal pumping rates were significantly reduced in 1–100 μM OBS groups compared to the DMSO control group (Figure 1A). OBS exposure decreased frequency of head swings and body bends in a concentration-dependent manner (Figure 1B,C). 100 μM OBS significantly reduced pharyngeal pumping rate (29.8%) and head swing frequency (23.4%), surpassing the effects of PFOS (Figure 1D,E). Both OBS and PFOS led to a significant decrease in the frequency of body bends, with OBS causing a reduction of about 50%, comparable to the decrease seen with PFOS (Figure 1F).

### 3.2. Distinct Effects of PFOS/OBS Exposure on Specific Neurotransmitter Systems in C. elegans

**Figure 2:**
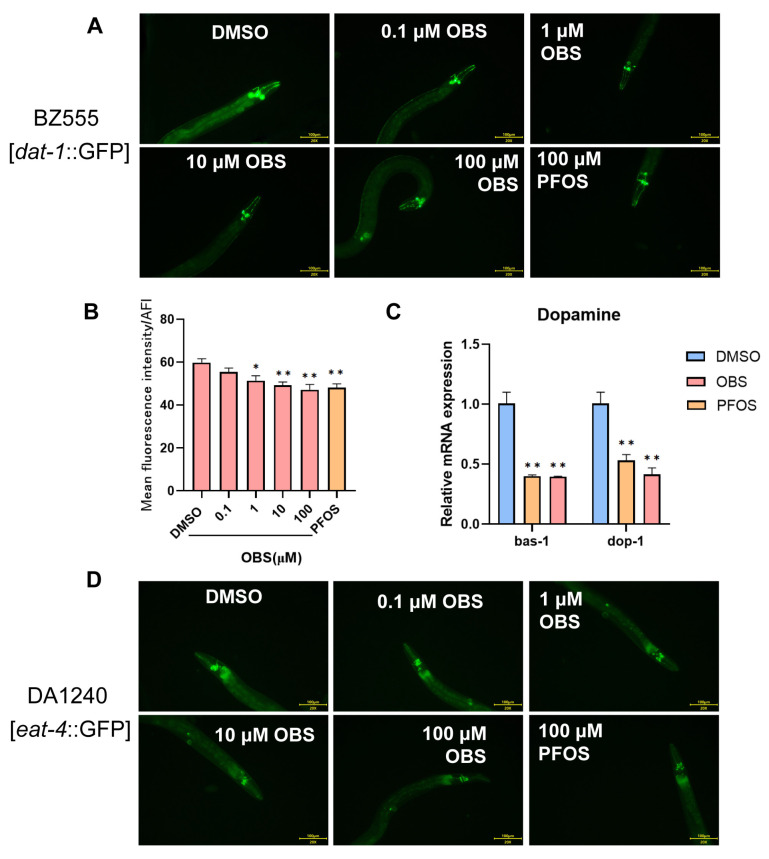

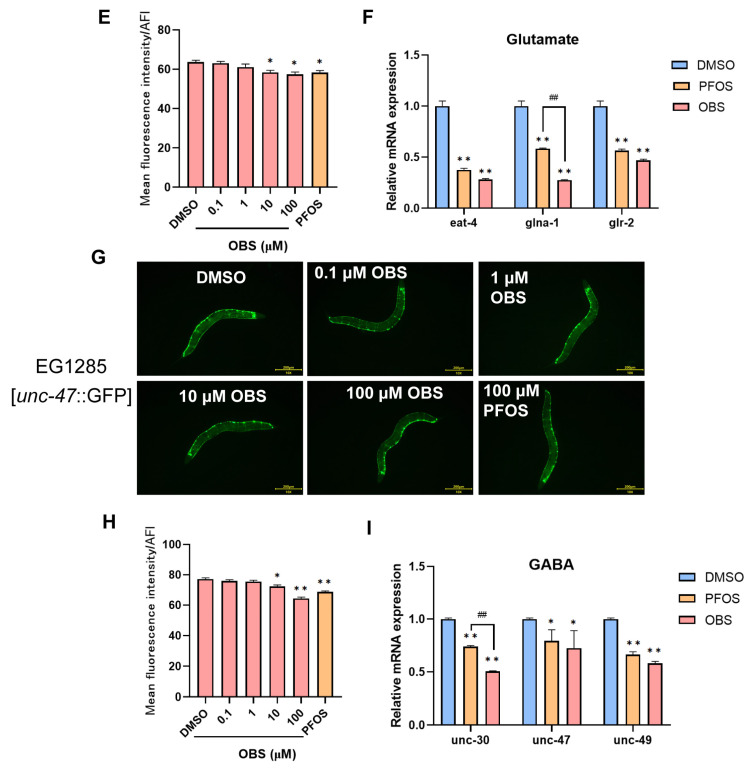
Effects of PFOS/OBS exposure on neuronal expression in transgenic *C. elegans*. (**A**,**D**,**G**) Representative fluorescence images were shown in transgenic *C. elegans.* (**B**,**E**,**H**) Relative fluorescent intensities were determined in transgenic *C. elegans*. Scale bar: 100 µm, *n* = 20. (**C**,**F**,**I**) Relative mRNA expression levels were examined by RT-qPCR. * *p* < 0.05, ** *p* < 0.01 vs. DMSO control; ^##^ *p* < 0.01 vs. PFOS, *n* = 15.

We utilized three transgenic strains (BZ555, DA1240, and EG1285) to specifically target and visualize different types of neurons (dopaminergic, glutamatergic, and GABAergic, respectively) because these neurons play crucial roles in neurobehavioral changes and neurotoxicity. We observed GFP-labelled fluorescence of neurons (Figure 2A,D,G) and measured the relative fluorescence intensity in transgenic worms, by which damage to these neurons can be quantified. The results showed that exposure to OBS (1, 10, 100 μM) significantly reduced the fluorescence intensity of dopaminergic neurons, similar to PFOS (Figure 2B). The genes *bas-1* and *dop-1* play critical roles in the synthesis and reception of dopamine, ensuring the proper functioning of dopaminergic neurons and the overall dopaminergic neurotransmission in the nervous system. We note that 100 μM OBS significantly caused approximately a 50% decrease in relative mRNA levels of *bas-1* and *dop-1*, similar with the effects of PFOS (Figure 2C).

Compared to the control group, exposure to OBS concentrations of 10–100 μM significantly decreased the fluorescence intensities of glutamatergic and GABAergic neurons (Figure 2E,H). The genes *eat-4*, *glna-1*, and *glr-2* are all related to the function and regulation of glutamatergic neurons in *C. elegans*. RT-qPCR confirmed the decrease in mRNA levels of *eat-4*, *glna-1* and *glr-2* in both PFOS and OBS exposed worms. Notably, *glna-1* was significantly down-regulated in OBS group compared to PFOS group (Figure 2F). The genes *unc-30*, *unc-47,* and *unc-49* play critical roles in the synthesis, transport, and reception of GABA, ensuring the proper functioning of GABAergic neurons and the overall inhibitory neurotransmission in the nervous system. We note that *unc-30* was expressed significantly lower in OBS group than PFOS group. Other two genes *unc-47* and *unc-49* were down-regulated in both OBS and PFOS groups (Figure 2I). These results indicate that OBS has damaging effects on neuronal expression, leading to abnormal levels of neurotransmission-related genes in *C. elegans*, similar with PFOS. In addition, the impacts of OBS on the expression of certain genes such as *glna-1* and *unc-30* are more pronounced than that of PFOS.

### 3.3. Distinct Effects of PFOS/OBS Exposure on Oxidative Stress in C. elegans

**Figure 3:**
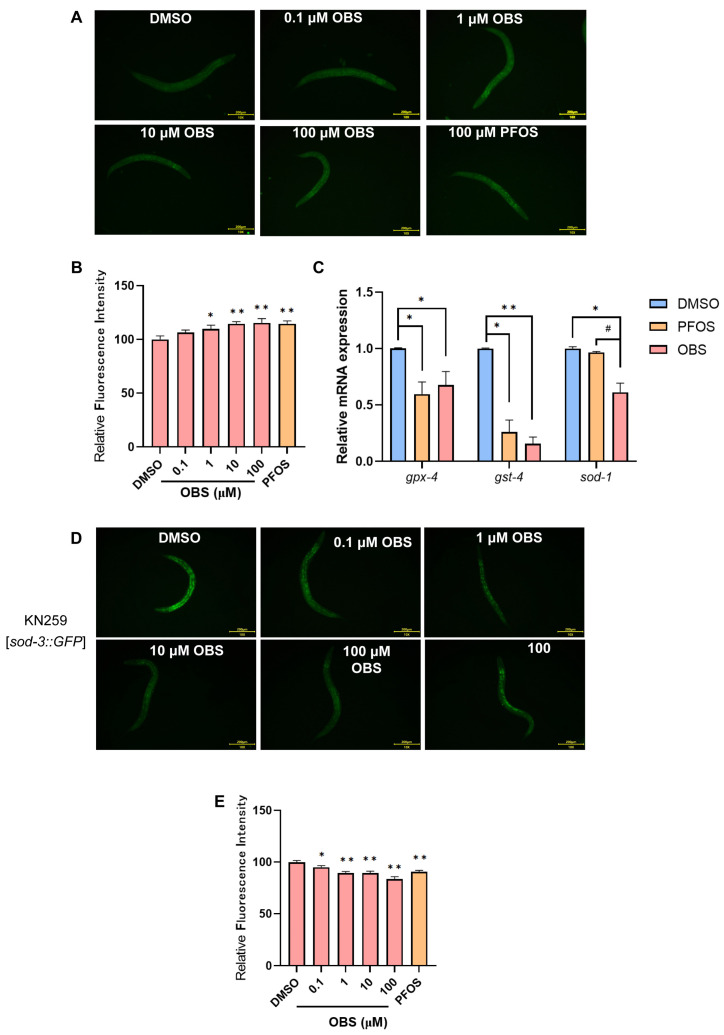
Effects of PFOS/OBS exposure on ROS and antioxidant genes in C. elegans. (**A**) Representative fluorescence images in N2 worms labelled by H2DCFDA fluorescent probe. (**B**) Relative fluorescent intensities indicate ROS production. (**C**) Relative expression levels of antioxidant genes were detected by RT-qPCR. (**D**) Representative fluorescence images were shown in KN259 transgenic C elegans. (**E**) Relative fluorescent intensities were determined in transgenic C elegans. * *p* < 0.05, ** *p* < 0.01 vs. DMSO control; ^#^ *p* < 0.05 vs. PFOS, scale bar: 200 µm, *n* = 15.

Oxidative stress induces the overproduction of ROS in *C. elegans*, overwhelming its antioxidant defenses, thereby causing oxidative damage, neurotoxicity, and impaired locomotor behaviors. To investigate the effects of OBS exposure on ROS levels in C elegans, we employed the H2DCFDA fluorescent probe (Nanjing Jiancheng Bioengineering Institute, Nanjing, China) to stain the C elegans. Compared with the DMSO group, the average fluorescence intensity significantly increased in a dose-dependent manner with the elevation of PFOS and OBS exposure concentrations (Figure 3A,B). A total of 100 μM PFOS and OBS remarkably decreased the expression of antioxidants, including *gpx-4*, *gst-4*, and *sod-1* in *C. elegans*. Moreover, the reduction in *sod-1* expression was more significant in *C. elegans* exposed to 100 μM OBS than in those exposed to PFOS (Figure 3C).

The KN259 (sod-3p::GFP) strain is a genetically engineered *C. elegans* strain used as a reporter for oxidative stress, providing a visual and quantitative means to assess the cellular response to oxidative stress. As shown in Figure 3D,E, the fluorescence intensities were significantly reduced in 0.1–100 μM OBS groups and the 100 μM PFOS group compared to the control group.

### 3.4. Distinct Effects of PFOS/OBS on Oxidative Stress-Neurotransmitter Regulatory Network

**Figure 4:**
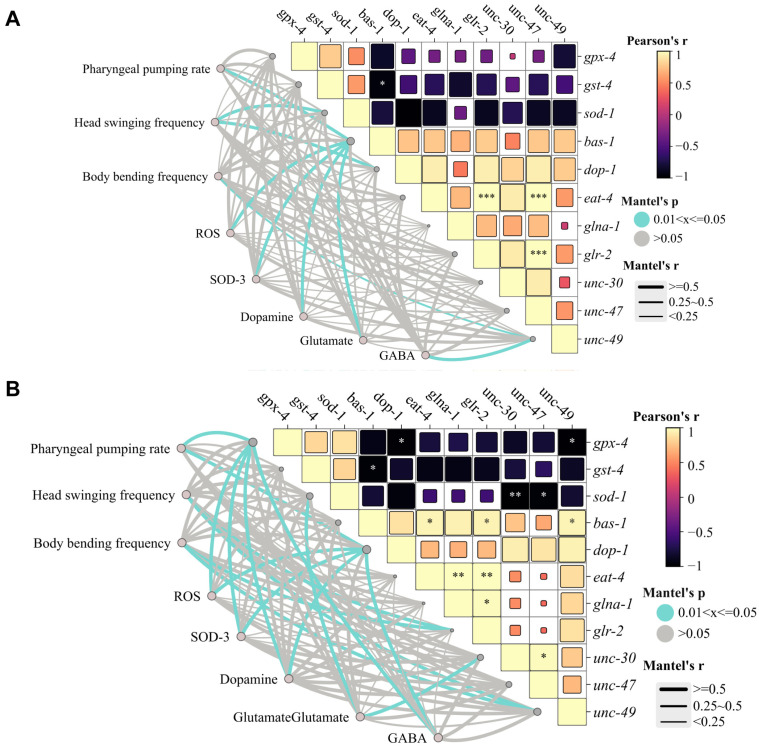
PFOS/OBS affect oxidative stress-neurotransmitter regulatory network. (**A**) PFOS exposure. (**B**) OBS exposure. Correlation between behaviors and neuronal development of *C. elegans* exposed to PFOS or OBS. The correlation coefficients (r and *p* value) were obtained from Pearson correlation analysis. A yellow color indicates a positive correlation, and a black color indicates a negative correlation. * *p* < 0.05, ** *p* < 0.01 *** *p* < 0.001.

We conducted an in-depth correlation network analysis. In terms of PFOS exposure, pharyngeal pumping rate is significantly positively correlated with the activity of dopaminergic neurons and ROS levels (*p* ≤ 0.05), suggesting that dysfunction of the dopaminergic system and oxidative stress jointly mediate the inhibition of feeding behavior. Motor behavior indices (frequency of head swinging and body bending) show moderate correlation with glutamate and GABAergic neurons (0.25 ≤ r < 0.5, *p* ≤ 0.05), confirming that motor coordination is regulated by the dynamic balance of excitatory/inhibitory neurotransmitters. Moreover, the expression of SOD-3 protein and *gpx-4* shows a synergistic downregulation trend, pointing to the collapse of the antioxidant defense system as the core mechanism of neurobehavioral damage. The downregulation of *bas-1* and *dop-1* in the dopaminergic system is consistent with the decreased fluorescence of dopaminergic neurons (0.25 ≤ r < 0.5) and is significantly correlated with the inhibition of pharyngeal pumping rate (*p* ≤ 0.05), confirming that impaired dopamine synthesis and signaling lead to feeding disorders. In the glutamatergic system, the suppression of *glr-2* expression is correlated with the decreased frequency of head swinging and body bending (0.25 ≤ r < 0.5, *p* ≤ 0.05). The inhibition of antioxidant genes (*sod-1*, *gpx-4*, *gst-4*) forms a positive feedback loop with ROS accumulation (*p* ≤ 0.05), further exacerbating the damage to dopaminergic and glutamatergic neurons (Figure 4A).

Compared with PFOS, OBS exhibits more significant targeting of neurotransmitter systems. The integrated analysis indicates that OBS induces neurotoxicity through the following pathways: inhibiting the SOD-3 and glutathione system (*gpx-4*, *sod-1*), leading to ROS accumulation and oxidative stress; specifically targeting neuronal dopaminergic (*dop-1*), GABAergic (*unc-30*, *unc-49*), and glutamatergic systems (*glr-2*), resulting in neurotransmitter disorders and ultimately manifesting as abnormal neurobehavioral functions, with differences in toxic mechanisms compared to PFOS (Figure 4B).

### 3.5. Binding Potential of PFOS and OBS with PMK-1 Protein

**Figure 5:**
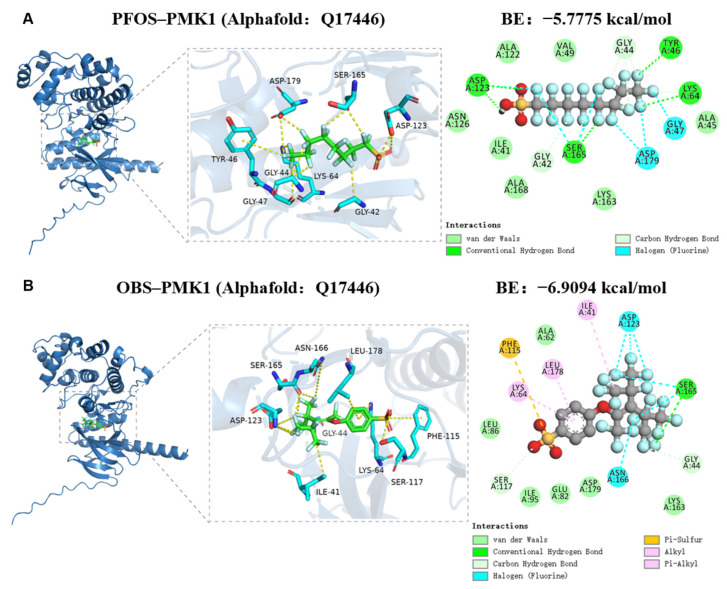

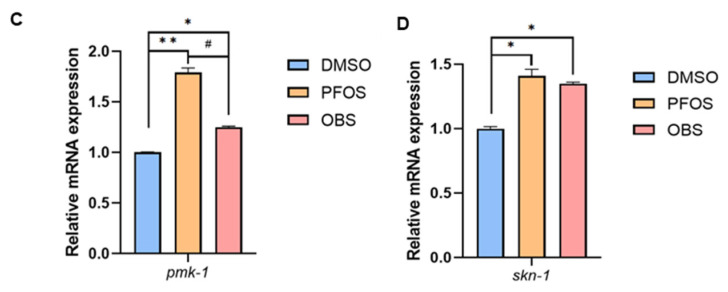
Binding potential of PFOS and OBS with PMK-1 protein. (**A**) Molecular docking between PFOS and PMK-1 protein. (**B**) Molecular docking between OBS and PMK-1 protein. (**C**,**D**) Relative expression levels of *pmk-1* and *skn-1* in N2 C elegans. * *p* < 0.05, ** *p* < 0.01 vs. DMSO, ^#^ *p* < 0.05 vs. PFOS; *n* = 15.

We employed molecular docking to further analyze the interaction between PFOS/OBS and the oxidative stress-related protein PMK-1. PFOS forms hydrogen bonds with residues Asp123, Ser165, Tyr46, and Lys64 on the PMK-1 receptor, and hydrophobic interactions with residues Gly44 and Gly42. Additionally, residues Asp179, Gly47, and Ser165 form halogen bonds with PFOS (Figure 5A). On the PMK-1 receptor, Ser165 interacts via hydrogen bonding with OBS, while residues Ile41, Leu178, and Lys64 interact hydrophobically with OBS. Furthermore, Phe115 on the protein interacts with OBS through a Pi-Sulfur interaction, and residues Ser117 and Gly44 form hydrophobic interactions with this compound. Residues Asn166, Asp123, and Ser165 also form halogen bonds with OBS (Figure 5B). OBS exhibits a higher binding affinity (−6.9094 kcal/mol) compared to PFOS (−5.7775 kcal/mol). Further, we found that PFOS/OBS significantly resulted in the increase in *pmk-1* and *skn-1* expression in N2 worms (Figure 5C,D). These findings suggest that both OBS and PFOS might modulate the antioxidant defense mechanisms in C elegans by influencing the p38 MAPK signaling pathway.

### 3.6. Distinct Effects of PFOS/OBS Exposure on Neurotoxicity in pmk-1 Mutant

**Figure 6:**
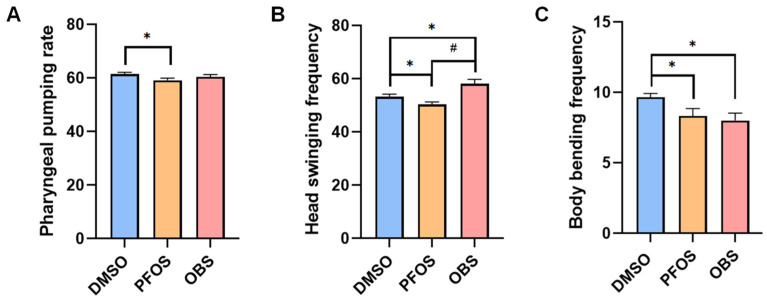
Effects of PFOS/OBS exposure on neurobehavioral activity in *pmk-1* mutant. (**A**) Pharyngeal pumping rate. (**B**) Head swinging frequency. (**C**) Body bending frequency; * *p* < 0.05 vs. DMSO, ^#^ *p* < 0.05 vs. PFOS; *n* = 15.

KU25 [*pmk-1 (km25)*] is *pmk-1* mutant of *C. elegans* with loss of function on PMK-1 protein, thereby often used for interfering the activity of the p38 MAPK signaling pathway. We compared the effects of 100 μM PFOS/OBS exposure on neurobehavioral activity, neurotransmission and oxidative stress in KU25 worms. The results showed that OBS had no significant decrease in the pharyngeal pumping rate compared to the control, but PFOS still reduced pharyngeal pumping rate (Figure 6A). PFOS significantly decreased frequency of head thrashes, whereas OBS significantly increased frequency of head thrashes in KU25 worms (Figure 6B). PFOS/OBS significantly induced the reduction in body bending frequency in KU25 worms (Figure 6C). These results showed that *pmk-1* mutation affected the alterations of pharyngeal pumping rate and frequency of head thrashes induced by OBS, but not PFOS.

**Figure 7:**
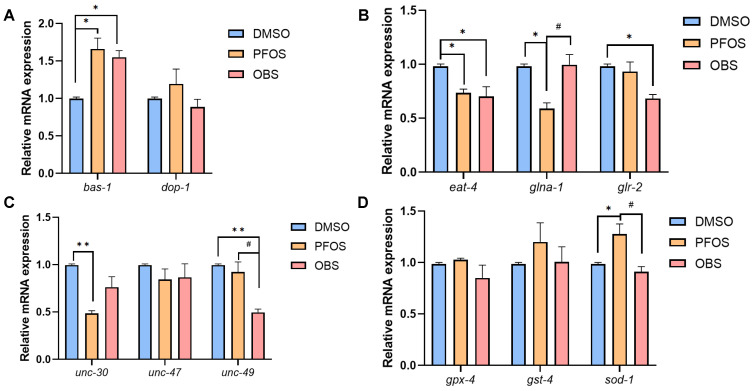
Effects of PFOS/OBS exposure on neurotransmission related genes and antioxidant genes in *pmk-1* mutants. (**A**–**C**) Relative mRNA expression levels of neuron related genes. (**D**) Relative mRNA expression levels of antioxidant genes. * *p* < 0.05, ** *p* < 0.01 vs. DMSO control; ^#^ *p* < 0.05 vs. PFOS; *n* = 15.

PFOS/OBS elevated *bas-1* expression, but did not affect *dop-1* expression in KU25 worms (Figure 7A), which were completely different from the decrease in dopamine neuron related genes in N2 worms. As for glutamate neuron related genes, OBS had no significant effects on *glna-1* gene expression while OBS still resulted in the decrease in *eat-4* and *glr-2* in KU25 worms (Figure 7B). Among GABA neuron related genes, *unc-30* and *unc-47* did not show obvious changes but *unc-49* was down-regulated by OBS in KU25 worms (Figure 7C). Similarly, OBS had no significant effects on ROS levels and antioxidant gene expression in KU25 worms compared to the control, although PFOS increased *sod-1* expression level (Figure 7D). These results showed that *pmk-1* mutation completely affected the alterations of dopamine neuron related genes and antioxidant genes when exposure to PFOS/OBS, while glutamate and GABA neuron-related genes showed different alterations in KU25 worms.

## 4. Discussion

PFOS is a widely used per-fluorinated compound known for its environmental persistence and neurotoxic effects. As a result, there has been a significant effort to develop and assess alternatives to PFOS such as OBS. The present study at the first time demonstrates that OBS significantly decreased pharyngeal pumping rate, head thrashes frequency and body bends frequency in *C. elegans*, indicating detrimental effects of OBS on neurobehavioral activity. OBS caused damage to neurons including dopaminergic, glutamatergic and GABAergic, and disrupted specific neurotransmitter systems. Additionally, OBS induced oxidative stress, modulating the antioxidant defense mechanisms in C elegans by influencing the p38 MAPK signaling pathway. OBS appears to have a more potent disruptive effect on neurobehaviors and key gene expression than PFOS, especially in *pmk-1* mutant worms. These effects may account for the greater neurotoxicity exhibited by OBS in contrast to PFOS. These findings suggest the pivotal role of the p38 MAPK pathway in OBS neurotoxicity and highlight the importance of thoroughly assessing the safety of OBS as a substitute for PFOS.

The pharynx of *C. elegans* is a glutamatergic neuron-regulated neuromuscular pump that connects the oral cavity to the intestine and, thus, serves as a sensitive indicator of neurotoxicity [33]. Our results showed that both OBS and PFOS significantly inhibited the pharyngeal pumping rate in worms, with OBS exhibiting stronger inhibition. OBS and PFOS may disrupt glutamatergic synaptic transmission, thereby impairing pharyngeal neuromuscular control. Additionally, exposure to OBS and PFOS led to abnormal movement behaviors, evidenced by a decrease in frequency of head thrashing and body bending, consistent with established PFAS-induced motor neuron dysfunction. Cross-species validation confirms these effects: 32 µg/L OBS induces depression-like behavior, social deficits, and memory impairment in zebrafish [34], while 20 µM PFOS inhibits larval swimming [35]; similarly, developing zebrafish exposed to PFOS and OBS exhibited impaired motor behaviors [36]. In rodents, PFOS (10.75 mg/kg, 3 months) impairs spatial memory [36] and lactational exposure disrupts offspring motor coordination [37]. In *C. elegans*, 20 µM PFOS reduces forward movement, body bends, and head thrashing [38]. These findings demonstrate that OBS and PFOS elicit cross-species neurobehavioral toxicity by interfering with glutamatergic signaling, with OBS exhibiting more potent disruption of neuromuscular control.

The present study systematically assessed the neurotoxic effects and underlying mechanisms of OBS using various strains of *C. elegans*. BZ555 (dat-1p::GFP, dopaminergic neuron marker) strain expresses GFP under the control of the *dat-1* promoter, which is specific to dopaminergic neurons [39]. Dopaminergic neurons are crucial for various behaviors and are often implicated in neurodegenerative diseases such as Parkinson’s disease [40]. DA1240 (eat-4::sGFP, glutamatergic neuron marker) strain expresses super GFP (sGFP) under the control of the eat-4 promoter, which is specific to glutamatergic neurons [41]. Glutamatergic neurons are involved in excitatory neurotransmission and play a key role in various cognitive functions [42]. EG1285 (unc-47p::GFP, GABAergic neuron marker) strain expresses GFP under the control of the *unc-47* promoter, which is specific to GABAergic neurons [43]. GABAergic neurons are involved in inhibitory neurotransmission and are essential for maintaining balance in neural activity [44]. In terms of neuronal development, OBS (10–100 μM) and PFOS (100 μM) markedly reduced fluorescence intensity in dopamine, glutamatergic, and GABAergic neurons, suggesting induction of neurodegenerative changes. RT-qPCR analysis further confirmed widespread downregulation of dopamine (*bas-1*, *dop-1*), glutamate (*eat-4*, *glna-1*, *glr-2*), and GABA (*unc-30*, *unc-47*, *unc-49*)-related genes with exposure to OBS and PFOS, with OBS showing more pronounced inhibitory effects. It is reported that exposure to PFOS and OBS reduced numbers of subcutaneous dopaminergic neurons in developing zebrafish [37] and 32 µg/L OBS disrupted GABA and glutamate homeostasis [38]. These findings are consistent with our results, where both PFOS and OBS caused neuronal damage, reduced neurotransmitter levels, and consequently led to behavioral impairments.

Oxidative stress, characterized by excessive ROS accumulation, disrupts antioxidant defenses in *C. elegans*, leading to oxidative damage and neurotoxicity. The two antioxidant enzymes, SOD and GST, are critical for ROS neutralization. Our findings demonstrate that both OBS and PFOS significantly inhibit the expression of antioxidant genes (*sod-3* and *gst-4*), impairing ROS clearance and exacerbating neuronal damage. This aligns with prior evidence: PFOS elevates lipid peroxidation and SOD activity while suppressing glutathione peroxidase activity [45], and 32 µg/L OBS induces mitochondrial dysfunction and ROS overproduction in zebrafish [46]. The resulting oxidative imbalance directly contributes to locomotor deficits, highlighting ROS-mediated neurotoxicity as a shared mechanism of PFAS-induced behavioral impairment across species.

Functional loss-of-function assays targeting the p38 MAPK pathway (via *pmk-1* mutation) revealed its essential role in mediating OBS- and PFOS-induced neurotoxicity in *C. elegans*. Molecular docking demonstrated that both compounds directly interact with critical PMK-1 residues (e.g., Asp123, Ser165), impairing kinase activity and disrupting downstream stress responses and neuroprotective mechanisms. In *pmk-1* mutant worms, the neurotoxic effects of OBS and PFOS were attenuated differently and markedly, confirming the regulatory role of p38 MAPK pathway. Consistently, RT-qPCR analysis showed that *pmk-1* mutation abolished transcriptional responses in antioxidant (*sod-3*, *gst-4*) and neurotransmitter-related genes, confirming *pmk-1* as a pivotal molecular target for neurotoxicity induced by PFOS and OBS in *C. elegans. pmk-1* mutation completely affected the alterations of dopaminergic genes and antioxidant genes, while glutamatergic, and γ-aminobutyric acid related genes showed different alterations when exposure to PFOS/OBS. These findings suggest that the distinct mechanisms in neurotransmitter imbalance of PFOS/OBS via p38 MAPK pathway.

Approximately 60–80% of human disease genes have orthologs in *C. elegans*, enabling direct translation to disease mechanism research and robust cross-species mechanistic extrapolation [24]. Critically, mutation of *pmk-1* (the ortholog of mammalian p38 MAPK) significantly alleviated the neurotoxic effects of OBS and PFOS in *C. elegans*, underscoring the conserved role of *pmk-1* mediated pathway in regulating oxidative stress and neuroinflammation that implicated in human neurodegenerative disorders such as Alzheimer’s and Parkinson’s diseases [47]. Evolutionary conservation extends to the antioxidant enzymes and neurotransmitter systems, and various human homologs are implicated in neurological disorders (Table S2) [48,49,50,51,52,53,54,55,56,57,58,59]. For example, the dopamine-synthetic *bas-1* (human DDC ortholog) links to Parkinson’s pathology [45]. The *unc-47* gene, homologous to human GAD1, encodes a key enzyme for GABA synthesis, and its dysregulation is associated with neurodegenerative diseases, epilepsy and autism [34]. Notably, downregulation of *eat-4* (vesicular glutamate transporter) may directly reduce glutamate release and impair neuromuscular control of pharyngeal pumping [46], whereas decreased *unc-30* expression may disrupt GABAergic inhibition, potentially contributing to motor behavioral abnormalities [48]. These findings highlight *C. elegans* as a powerful translational model for dissecting PFAS neurotoxicity mechanisms, paving the way for targeted therapeutic interventions in humans.

## 5. Conclusions

This study systematically evaluates the neurotoxic mechanisms of PFOS and its alternative OBS in *C. elegans*, demonstrating that both compounds induce locomotor dysfunction, neuronal structural damage, and disruption of dopaminergic, glutamatergic, and GABAergic pathways. Loss-of-function analysis and molecular docking experiment confirm that the p38 MAPK pathway plays a critical role in mediating oxidative stress responses to PFOS/OBS, with OBS showing stronger binding affinity to PMK-1 than PFOS, suggesting a higher neurotoxic potential. By integrating oxidative stress, neurotransmitter dysregulation, and p38 MAPK pathway activation into a cohesive mechanistic framework, our findings highlight the necessity of cautious evaluation and regulation of PFOS alternatives, as their neurotoxic risks may be comparable to or even exceed those of PFOS, thereby emphasizing the importance of thorough safety assessments to protect environmental and public health.

## Data Availability

Data will be made available on request.

## Supplementary Materials

The following supporting information can be downloaded at: https://www.mdpi.com/article/10.3390/toxics13080662/s1, Table S1: RT-qPCR primer sequence. Table S2: Comparison of genetic homology between *C. elegans* and Homo sapiens.

## Abbreviations

The following abbreviations are used in this manuscript:

OBS	Sodium p-perfluorous nonenoxybenzenesulfonate
PFOS	Perfluorooctanesulfonic acid
PFASs	Perfluoroalkyl and polyfluoroalkyl substances
*C. elegans*	*Caenorhabditis elegans*
GFP	Green fluorescent protein
DA	Dopamine
Glu	Glutamate
GABA	Gamma-aminobutyric acid
Ach	Acetylcholine
AD	Alzheimer’s disease
PD	Parkinson’s disease
MAPK	Mitogen-activated protein kinase
ROS	Reactive oxygen species
*E. coli*	*Escherichia coli*
NGM	Nematode growth medium
DMSO	Dimethyl sulfoxide
SOD	Superoxide dismutase
GST	Glutathione S-transferase
