# Supplementary material for: Distinct Effects of PFOS and OBS on Neurotoxicity via PMK-1 Mediated Pathway in Caenorhabditis elegans
Source: Toxics. 2025 Aug 6;13(8):662. doi: 10.3390/toxics13080662 (PMC12390093; doi:10.3390/toxics13080662)
Supplement: Supplementary file 1 [file toxics-13-00662-s001.zip › toxics-3784380-supplementary.pdf]

Table. S1 RT-qPCR primer sequence

**Gene name**	**Forward sequence (5'–3')**	**Reverse sequence (5'–3')**
tba-1	TCGGTAATGCTTGCTGGGAG	CTCCATCGGCTTGTTGGTCT
bas-1	TCACATGGAAATCGTGCCCA	GCTTCGTCGTCCACCTTGTT
dop-1	GACATCACCACATGCACAGC	CGACGGTTGCAGTACCTGAT
eat-4	TGCCGGATTAACTTGCCCAT	GTCTCAGCAGCTCCGTATCC
glna-1	TGGTCCCATGTTGATCTCGG	GATGAAGCGATGCAAGGTCG
glr-2	TCTCTTCATACACGGCTAAT	CCTTCATTGACACCATACAG
unc-30	GCAGCAGCAGTCGCATAATC	GGGTTAATGGTGCGAGGGAA
unc-47	ACGTTTGCTACTGGACAGGG	CCATCCGGCTTTGTCAACAC
unc-49	CGTACTCCTCTCCGCACATC	ATTCCAACGTCGACTGGCTT
gpx-4	TACTTACTTTGGCTGTTTCTTTCACC	TTTCCAGCGCAGAGTATCG
gst-4	GCTCAATGTGCCTTACGAGG	CAGCAATCACAATATCAGCCCA
sod-1	CGTAGGCGATCTAGGAAATGTG	TGACGAGCGTGTCGGTGAG
sod-3	TGCAATCTACTGCTCGCACT	CTGGGAGAGTGTGCTTGGAG
pmk-1	CTGGATACGTGGCAACAAGA	CATCGTGATAAGCAGCCAGA
skn-1	CCCAACATCCAACTACGCCT	AAATCTGGAACGCCGACACT

Table. S2 Comparison of genetic homology between *C. elegans* and Homo sapiens

**Function**	***C. elegans***	**Homo sapiens**	**Identity%**	**Link to human neuropsychiatric disorders**	**Ref**
**Oxidative stress**	*gpx-4*	GPX-4	33.87	Amyotrophic lateral sclerosis	[49]
	sod-1	SOD1	57.52	Amyotrophic lateral sclerosis, Alzheimer's disease	[50]
	sod-3	SOD2	61.47	Alzheimer's disease	[51]
	gst-4	GSTM4	26.80	Alzheimer's disease, Parkinson's disease	[52]
**Dopaminergic**	*bas-1*	DDC	45.54	Parkinson's disease, Depression	[53]
	dop-1	DRD1	37.70	Alzheimer's disease, Parkinson's disease	[54]
**Glutamatergic**	*eat-4*	SLC17A8	45.96	Alzheimer's disease, Seizures	[55]
	glna-1	GLS2	44.60	Seizures	[56]
	glr-2	GRIN2B	36.92	Alzheimer's disease	[57]
**GABAergic**	*unc-30*	PITX2	31.62	Amyotrophic lateral sclerosis, Parkinson's disease	[48,58]
	unc-47	SLC32A1	36.85	Neurodegenerative diseases, Seizures	[53]
	unc-49	GABRG2	35.57	Seizures, Depression, Anxiety disorders	[59]
